# GADD45β Regulates Hepatic Gluconeogenesis via Modulating the Protein Stability of FoxO1

**DOI:** 10.3390/biomedicines9010050

**Published:** 2021-01-08

**Authors:** Hyunmi Kim, Da Som Lee, Tae Hyeon An, Tae-Jun Park, Eun-Woo Lee, Baek Soo Han, Won Kon Kim, Chul-Ho Lee, Sang Chul Lee, Kyoung-Jin Oh, Kwang-Hee Bae

**Affiliations:** 1 Metabolic Regulation Research Center, Korea Research Institute of Bioscience and Biotechnology (KRIBB), Daejeon 34141, Korea; khm7607@kribb.re.kr (H.K.); dasom89@kribb.re.kr (D.S.L.); anth0291@kribb.re.kr (T.H.A.); tjpark@kribb.re.kr (T.-J.P.); ewlee@kribb.re.kr (E.-W.L.); bshan@kribb.re.kr (B.S.H.); wkkim@kribb.re.kr (W.K.K.); lesach@kribb.re.kr (S.C.L.); 2 Department of Functional Genomics, KRIBB School of Bioscience, Korea University of Science and Technology (UST), Daejeon 34141, Korea; 3 Laboratory Animal Resource Center, Korea Research Institute of Bioscience and Biotechnology (KRIBB), Daejeon 34141, Korea; chullee@kribb.re.kr

**Keywords:** GADD45β, gluconeogenesis, FoxO1, protein stability, cAMP signaling

## Abstract

Increased hepatic gluconeogenesis is one of the main contributors to the development of type 2 diabetes. Recently, it has been reported that growth arrest and DNA damage-inducible 45 beta (GADD45β) is induced under both fasting and high-fat diet (HFD) conditions that stimulate hepatic gluconeogenesis. Here, this study aimed to establish the molecular mechanisms underlying the novel role of GADD45β in hepatic gluconeogenesis. Both whole-body knockout (KO) mice and adenovirus-mediated knockdown (KD) mice of GADD45β exhibited decreased hepatic gluconeogenic gene expression concomitant with reduced blood glucose levels under fasting and HFD conditions, but showed a more pronounced effect in GADD45β KD mice. Further, in primary hepatocytes, GADD45β KD reduced glucose output, whereas GADD45β overexpression increased it. Mechanistically, GADD45β did not affect Akt-mediated forkhead box protein O1 (FoxO1) phosphorylation and forskolin-induced cAMP response element-binding protein (CREB) phosphorylation. Rather it increased FoxO1 transcriptional activity via enhanced protein stability of FoxO1. Further, GADD45β colocalized and physically interacted with FoxO1. Additionally, GADD45β deficiency potentiated insulin-mediated suppression of hepatic gluconeogenic genes, and it were impeded by the restoration of GADD45β expression. Our finding demonstrates GADD45β as a novel and essential regulator of hepatic gluconeogenesis. It will provide a deeper understanding of the FoxO1-mediated gluconeogenesis.

## 1. Introduction

Gluconeogenesis, the de novo glucose synthesis, is important in maintaining blood glucose levels to meet the whole-body energy requirements during the state of energy exhaustion [1]. However, excessive gluconeogenesis significantly contributes to type 2 diabetes, which increases the risk of many complications, such as cardiovascular disease, kidney disease, and cancer [2].

Master transcription factors for regulating hepatic gluconeogenesis include cAMP response element-binding protein (CREB) and forkhead box protein O1 (FoxO1) [3]. They are mainly regulated by insulin and glucagon, the main counter regulatory hormones involved in balancing blood glucose levels [4]. Glucagon activates protein kinase A (PKA) by raising the cAMP levels and subsequently phosphorylates CREB, which induces the expression of gluconeogenic genes including glucose-6-phosphatase catalytic subunit (G6PC) and phosphoenolpyruvate carboxykinase-1 (PCK1) [5]. On the other hand, FoxO1 is negatively regulated by insulin [6]. Insulin inhibits FoxO1 activity by promoting the nuclear exclusion and cytoplasmic retention of FoxO1 via the AKT-mediated phosphorylation of FoxO1 at ser256 [7,8]. Phosphorylated FoxO1 is sequestered in the cytoplasm by binding to the 14-3-3 proteins [9,10,11], eventually becoming a target of the ubiquitin-mediated degradation [12]. FoxO1 can also be phosphorylated and acetylated by glucagon for regulating hepatic gluconeogenesis [5,13,14]. Additionally, glucagon can regulate FoxO1 protein stability and nuclear localization [14].

Growth arrest and DNA damage-inducible 45 beta (GADD45β) is a scaffold protein involved in DNA damage, apoptosis, and oxidative stress [15,16,17,18]. As a starvation response gene, GADD45β expression is increased 2 to 5 times by fasting in mouse liver [19]. Recently, it has been reported that fasting-induced hepatic GADD45β expression regulates hepatic lipid metabolism by inhibiting hepatic fatty acid uptake [20]. On the other hand, hepatic GADD45β is suppressed by signal transducer and activator of transcription 3 (STAT3), which inhibits gluconeogenesis, and is a direct transcriptional target of STAT3 [21]. The STAT3-mediated suppression of hepatic gluconeogenesis is associated with FoxO1 activity [22,23,24]. Therefore, we hypothesized GADD45β might be related with the regulation of hepatic gluconeogenesis.

Here, we demonstrated the role of GADD45β in hepatic gluconeogenesis by using the adenovirus-mediated GADD45β knockdown (KD) and GADD45β overexpression system as well as GADD45β knockout (KO) mice. Collectively, our findings described that GADD45β is an essential regulator of cAMP-induced hepatic gluconeogenesis in a FoxO1-dependent manner. This study will deepen understanding of the molecular mechanism of cAMP/PKA-induced and FoxO1-mediated gluconeogenesis.

## 2. Experimental Section

### 2.1. Animal Experiments

Eight-week-old male C57BL/6 mice were purchased from ORIENT BIO. Mice lacking the whole-body expression of GADD45β were obtained from Chul-Ho Lee’s Lab in the Korea Research Institute of Bioscience and Biotechnology (KRIBB). All mice were housed and maintained in a 12 h light/12 h dark cycle under temperature- and humidity-controlled conditions with free access to food and water. Mice were fed either a standard chow diet or an HFD (60 kcal % fat diet: D12492 of Research Diets) for 12 weeks. For all animal experiments involving adenoviruses, 10-week-old male C57BL/6 mice were tail vein-injected with Ad-shGADD45β or Ad-US (control) at 0.25–0.5 × 10^9^ pfu per mice. To induce fasting conditions, mice were fasted for 6 h, 16 h, or 24 h with free access to water. All animal procedures were approved by the Institutional Animal Care and Use Committee of the KRIBB (KRIBB-AEC-20155, 15 June 2020) and were performed according to the guidelines for the Care and Use of Laboratory Animals published by the US National Institutes of Health.

### 2.2. Plasmids and Recombinant Adenoviruses

The full-length sequences for mouse GADD45β, FoxO1, and FoxO1-ADA were reverse transcribed using liver RNA derived from the C57BL/6 mice. The sequences were amplified via PCR and inserted into the pcDNA3-Flag or pcDNA3-HA expression vector. The 6X-insulin response elements (IRE) and G6PC promoter sequences were amplified by PCR using genomic DNA from the C57BL/6 mice and inserted into the pGL4-luciferase reporter vector. Adenoviruses expressing GFP control, GADD45β, unscrambled nonspecific RNAi control (US), and shGADD45β were used as previously described [25]. For animal experiments, the adenoviruses were purified on a CsCl gradient, dialyzed against PBS buffer containing 10% glycerol, and stored at −80 °C.

### 2.3. Culture of Primary Hepatocytes

Primary hepatocytes were isolated from 8-week-old male C57BL/6 mice by collagenase perfusion method [25]. 1 × 10^6^ cells were plated in 6-well plates with medium 199 (Sigma-Aldrich, St Louis, MO, USA) supplemented by 10% FBS, 1% antibiotics, and 10 nM dexamethasone. After attachment, cells were infected with adenoviral vectors for 48 h or 72 h. Cells were maintained in medium 199 without 10% FBS for 16 to 18 h and then treated with 10 uM forskolin or 100 nM insulin.

### 2.4. Quantitative PCR

Total RNA from primary hepatocytes or mouse liver was extracted using the easy-spin Total RNA Extraction Kit (iNtRON Biotechnology, Seongnam, Korea); 2 μg of total RNA was reverse transcribed into cDNA with the M-MLV Reverse Transcriptase (Promega, Madison, WA, USA). The cDNA was analyzed by quantitative PCR using the SYBR green PCR kit in a C1000 Touch™ Thermal Cycler (Bio-Rad Laboratories, Hercules, CA, USA). All data were normalized to the expression of ribosomal L32 in the corresponding sample.

### 2.5. Glucose Production in Primary Hepatocytes

Glucose production was assayed, as previously described [25]. Primary hepatocytes were infected with adenoviral vectors and incubated in serum-free media for 16 h. The cells were then stimulated with 10 uM forskolin and 1 nM dexamethasone in the Krebs-Ringer Buffer (KRB) containing gluconeogenic substrates, 20 mM lactate, and 2 mM pyruvate, for 8 h. Glucose concentrations were measured using a Glucose Assay Kit (Cayman Chemical, Ann Arbor, MI, USA).

### 2.6. Seahorse Analysis

Mitochondrial functions, manifested as the oxygen consumption rate and extracellular acidification rate were determined with an XF24 extracellular flux analyzer (Seahorse Bioscience, North Billerica, MA, USA). The glycolysis in Ad-US or Ad-shGADD45β-infected primary hepatocytes was assessed by analyzing their respective extracellular acidification rate (ECAR). Hepatocytes were seeded on collagen-coated XF24 cell culture microplate 72 h before the experiment. The cells were maintained in a glucose-free culture medium. After the addition of 20 mM glucose, 2.5 uM oligomycin, and 50 mM 2-deoxyglucose, the ECAR rate was measured and normalized to the protein content in each sample. The fatty acid oxidation in Ad-US or Ad-shGADD45β-infected primary hepatocytes was assessed by analyzing their oxygen consumption rate (OCR). The OCRs were measured before and after the injection of BSA or 250 uM palmitate, 2.5 uM oligomycin, 10 uM fluoro-carbonyl-cyanide phenylhydrazone (FCCP), and 2 uM rotenone plus 5 uM antimycin A. The OCRs were normalized to the protein content in each sample.

### 2.7. Western Blot Analysis

The protein in the lysates of primary hepatocytes or mouse livers was resolved by SDS-PAGE and transferred to PVDF membranes. The membranes were incubated with the primary antibody against phospho-AKT (Ser473), phospho-AKT (Thr308), AKT, phospho-FoxO1 (Ser256), FoxO1, phospho-CREB (Ser133), CREB, phospho-AMPKα (Thr172), or AMPKα (Cell Signaling Technology, Danvers, MA, USA). Other membranes were incubated with the primary antibody against Flag-M2 (Sigma-Aldrich, St Louis, MO, USA), HA (Santa Cruz, CA, USA), or HSP90 (Santa Cruz, CA, USA). These were followed by an incubation with a horseradish peroxidase-conjugated secondary antibody (Santa Cruz, CA, USA) and were visualized using an enhanced chemiluminescence detection (GE Healthcare, Madison, WI, USA). Then the protein bands were quantified by ImageJ.

### 2.8. Cycloheximide Chase Assay

Mouse primary hepatocytes were treated with 25 ug/mL cycloheximide (CHX), a protein synthesis inhibitor, for the indicated time. The protein samples from cell lysates were subjected to Western blot analysis. Target protein bands were quantified by ImageJ.

### 2.9. Luciferase Assay

Human hepatoma HepG2 cells were maintained in Ham’s F12 medium supplemented with 10% FBS and 1% antibiotics and 200 ng of luciferase construct, 50 ng of β-galactosidase plasmid, or 1 to 25 ng of an expression vector containing GADD45β, FoxO1, or FoxO1-ADA were transfected into HepG2 cells with the TransIT-LT1 Reagent (Mirus Bio, Madison, WI, USA). Promoter activities were measured 48 h after transfection using a luciferase reporter assay system (Promega, Madison, WA, USA) and normalized to β-galactosidase levels.

### 2.10. Immunocytochemistry

HEK293 cells were maintained in Dulbecco’s modified Eagle medium (DMEM) supplemented with 10% FBS and 1% antibiotics. Cells were transfected with pcDNA3-GFP-GADD45β and pcDNA3-RFP-FoxO1. Cells were fixed and stained with DAPI. Images were captured with an Olympus DP30BW digital camera and processed using MetaMorph version 7.1 (Universal Imaging, Media, PA, USA).

### 2.11. Nuclear/Cytoplasmic Fractionation

Nuclear and cytoplasmic proteins were extracted from cells using the NE-PERTM Nuclear and Cytoplasmic Extraction Reagents (Thermo Fisher Scientific, Waltham, MA, USA).

### 2.12. Immunoprecipitation

Cells were lysed in five volumes of the lysates in lysis buffer, which consists of 1 M Tris, pH 7.5, 150 mM NaCl, 1 mM EGTA, pH 8.0, 1 mM EDTA, pH 8.0, 1% Triton X-100, 2.5 mM Na4P2O7, 50 mM NaF, 5 mM β-glycerol-phosphate, 1 mM Na3VO4, 1 mM DTT, and one tablet of complete protease inhibitor (Roche Diagnostics, Indianapolis, IN, USA). Equal amounts of protein from the lysates were incubated overnight at 4 °C with anti-flag M2 or anti-HA agarose antibody. The immunoprecipitates were washed three times with lysis buffer and eluted with a sample buffer without β-mercaptoethanol. The eluents were then prepared in a sample buffer, separated with SDS-PAGE, and analyzed by Western blot.

### 2.13. Statistical Analysis

Results were shown as mean ± standard deviation (SD) or mean ± standard error of the mean (SEM). The statistical differences between the two experimental groups were analyzed by the two-tailed unpaired Student’s *t*-test. Values of *p* < 0.05 were considered statistically significant.

## 3. Results

### 3.1. Hepatic GADD45β Deficiency Suppresses Hepatic Gluconeogenesis

**Figure 1:**
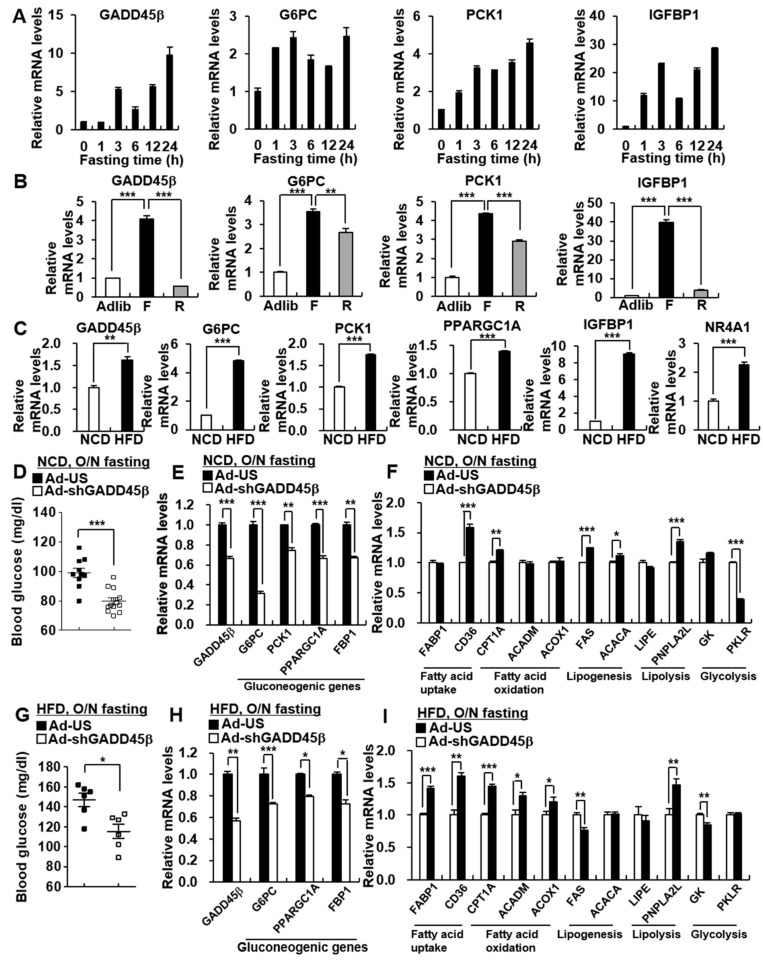
Knockdown of hepatic GADD45β suppresses hepatic gluconeogenic gene expression under fasting and high-fat diet conditions. (**A**) 8-week-old male C57BL/6 mice were fasted for the indicated time (*n* = 4/group). qPCR analysis showing the effects of fasting on mRNA levels of GADD45β, G6PC, PCK1, and IGFBP1 in livers. (**B**) qPCR analysis showing mRNA levels of GADD45β, G6PC, PCK1, and IGFBP1 in livers of 8-week-old C57BL/6 mice under ad libitum (Adlib), 24-h fasting (F), and 24-h refeeding after 24-h fasting (R) conditions (*n* = 7/group). (**C**) 8-week-old male C57BL/6 mice were fed a standard chow diet (NCD) or high-fat diet (HFD) for 12 weeks (*n* = 5/group). qPCR analysis showing the effects of HFD on mRNA levels of GADD45β, G6PC, PCK1, PPARGC1A, IGFBP1, and NR4A1 in the livers. (**D**–**F**) 8-week-old C57BL/6 male mice were infected with Ad-shGADD45β (*n* = 15) or Ad-US control (*n* = 10) for 7 days. Blood glucose levels after 16 h of fasting (**D**), qPCR analysis showing expression levels of GADD45β, gluconeogenic genes (**E**), and other metabolic genes (**F**). (**G**–**I**) 8-week-old C57BL/6 male mice were fed a HFD for 12 weeks and then were infected with Ad-shGADD45β (*n* = 6) or Ad-US control (*n* = 6) for 7 days. Blood glucose levels after 16 h of fasting (**G**), qPCR analysis showing expression levels of GADD45β, gluconeogenic genes (**H**), and other metabolic genes (**I**). Data in (**A**–**C**,**E**,**F**,**H**,**I**) represent the mean ± SD, and data in (**D**,**G**) represent the mean ± SEM (* *p* < 0.05; ** *p* < 0.005; *** *p* < 0.0005; *t*-test).

Hepatic gluconeogenesis is known to be enhanced under both fasting and HFD conditions. We found that whole-body GADD45β knockout (KO) mice exhibited decreased hepatic gluconeogenic gene expression and reduced blood glucose levels under fasting and HFD conditions (Figure S1A,B,D,E). Similarly to the previous report [20], there was decreased expression of fatty acid uptake genes such as CD36 in livers of whole-body GADD45β KO mice (Figure S1C,F). To access the relationship between hepatic GADD45β and gluconeogenesis, we observed hepatic GADD45β expression during fasting and HFD conditions (Figure 1A–C). Hepatic GADD45β expression was enhanced concomitant with increased expression of gluconeogenic genes, including G6PC, PCK1, and insulin-like growth factor binding protein-1 (IGFBP1) under fasting conditions (Figure 1A,B). Meanwhile, refeeding restored the fasting-induced expression of hepatic GADD45β and gluconeogenic genes (Figure 1B). Furthermore, a HFD feeding promoted the expression of hepatic GADD45β and gluconeogenic genes, including G6PC, PCK1, peroxisome proliferator-activated receptor gamma coactivator 1-alpha (PPARGC1A), IGFBP1, and orphan nuclear receptor 4A1 (NR4A1) (Figure 1C). Importantly, these data suggest that hepatic GADD45β would be involved in the hepatic gluconeogenic program.

To confirm the effects of liver-specific GADD45β deficiency on hepatic gluconeogenesis, we performed adenovirus-mediated knockdown (KD) of GADD45β in the liver under fasting and HFD conditions. Hepatic GADD45β KD was shown to suppress the expression of gluconeogenic genes such as G6PC, PCK1, PPARGC1A, and fructose-bisphosphatase 1 (FBP1) concomitant with decreased blood glucose levels under fasting conditions on both standard chow diet (NCD) and HFD (Figure 1D,E,G,H). These data suggest that hepatic GADD45β deficiency would suppress hepatic gluconeogenesis. Additionally, unlike whole-body GADD45β KO mice [20], hepatic GADD45β KD increased the expression of genes involved in FA uptake and FA oxidation, particularly under HFD conditions (Figure 1F,I). Similar to the whole-body KO mice [20], hepatic GADD45β KD slightly promoted triglyceride (TG) accumulation in the liver under HFD conditions (Figure S2A,B).

### 3.2. Hepatic GADD45β Regulates Glucose Production by Modulating Hepatic Gluconeogenesis

**Figure 2:**
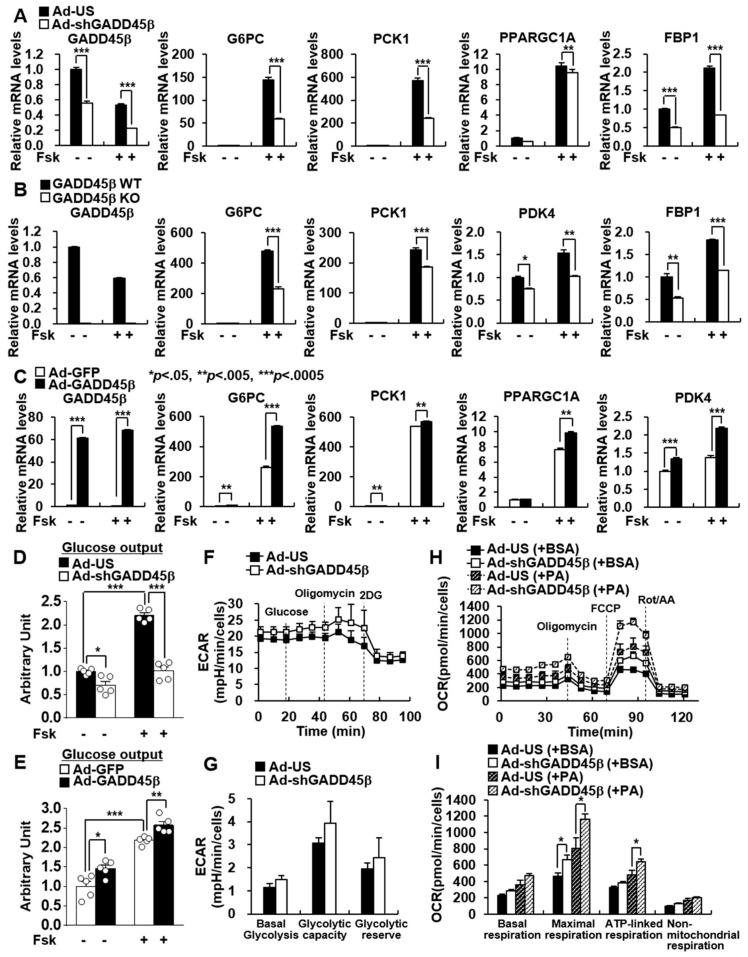
Hepatic GADD45β regulates glucose production by modulating gluconeogenesis. (**A**–**C**) qPCR analysis showing the effects of GADD45β knockdown (**A**), knockout (**B**), and overexpression (**C**) on expression levels of GADD45β and gluconeogenic genes. Mouse primary hepatocytes were infected with the Ad-US (control) or Ad-shGADD45β (knockdown) for 72 h (**A**) or with Ad-GFP or Ad-GADD45β for 48 h (**C**). Cells were treated with or without 10 uM Fsk for 2 h. (**D**) Glucose production in mouse primary hepatocytes infected with Ad-US or Ad-shGADD45β. (**E**) Glucose production in mouse primary hepatocytes infected with Ad-GFP or Ad-GADD45β. (**F**–**I**) Mouse primary hepatocytes were infected with Ad-US or Ad-shGADD45β. (**F**,**G**) Measurement of Glycolytic rate using extracellular acidification rate (ECAR). Cells were incubated with glucose, oligomycin, and 2DG. (**H**,**I**) Measurement of FA oxidation (FAO) using oxygen consumption rate (OCR). Cells were incubated with BSA or palmitate (PA), oligomycin, FCCP, rotenone, and antimycin A. Data in (**A**–**C**,**I**) represent the mean ± SD, and data in (**D**,**E**) represent the mean ± SEM (* *p* < 0.05; ** *p* < 0.005; *** *p* < 0.0005; *t*-test).

Stimulation of the cyclic adenosine monophosphate (cAMP) signaling pathway by forskolin (Fsk) mimics the fasting action of glucagon. In line with in vivo studies, GADD45β KD and KO reduced the basal and Fsk-mediated induction of gluconeogenic genes in primary hepatocytes (Figure 2A,B). Adenovirus-mediated expression of GADD45β promoted the basal and Fsk-induced expression of gluconeogenic genes (Figure 2C). As a result, hepatic GADD45β deficiency suppressed glucose output, whereas the overexpression of GADD45β promoted it (Figure 2D,E). These data suggest that GADD45β contributes to glucose production via hepatic gluconeogenesis.

Gluconeogenesis and glycolysis regulate each other reciprocally. Therefore, we observed the effects of GADD45β on glycolysis. However, depletion of hepatic GADD45β did not affect the key parameters of glycolytic activity-glycolysis, glycolytic capacity, and glycolytic reserve as indicated by the extracellular acidification rate (ECAR) (Figure 2F,G). Moreover, FA oxidation (FAO) was measured by assessing the changes in the oxygen consumption rate (OCR). GADD45β KD increased maximal respiration and ATP-linked respiration (Figure 2H,I). Further, it mildly promoted the basal and AICAR-induced phosphorylation levels of AMPK at Thr172 (Figure S2C).

### 3.3. GADD45β Enhances FoxO1 Protein Stability Rather Than AKT-Mediated FoxO1 Phosphorylation and Fsk-Induced CREB Phosphorylation

As shown above, GADD45β regulated Fsk-mediated induction of hepatic gluconeogenic genes and glucose production. Fsk has been reported to phosphorylate and activate protein kinase A (PKA)/CREB via cAMP and can also enhance FoxO1 protein levels [14]. Here, we confirmed that Fsk enhanced FoxO1 protein levels and CREB phosphorylation in mouse primary hepatocytes (Figure S3A,B). Phosphorylation of CREB at Ser133 peaked at 0.5 h after Fsk treatment, whereas FoxO1 protein expression was gradually increased until 3 h by Fsk, suggesting that FoxO1 protein expression might be necessary to sustain the effect of Fsk (Figure S3A,B).

**Figure 3:**
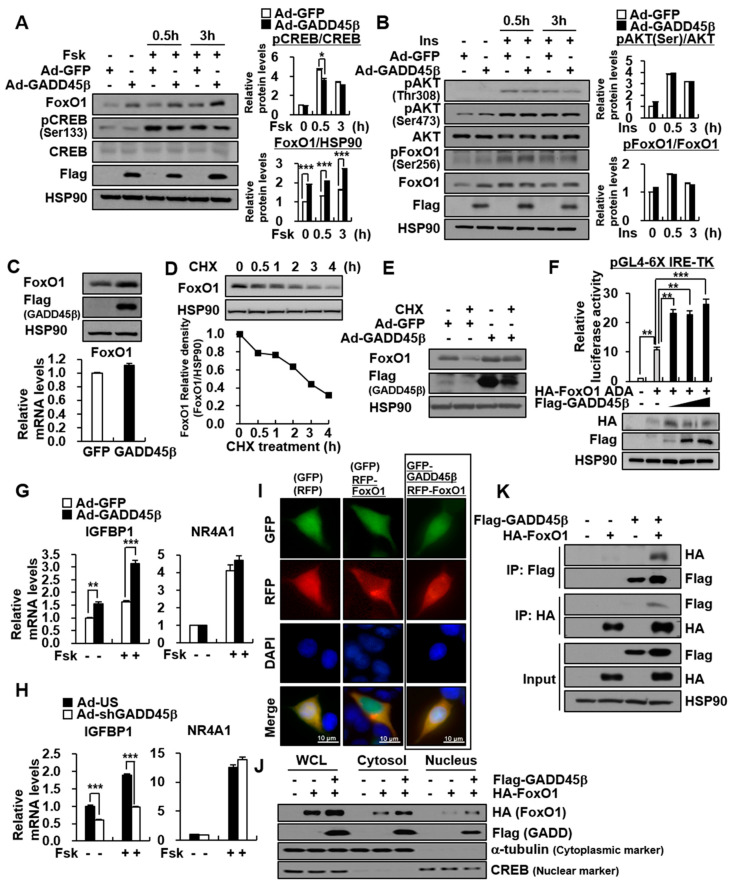
GADD45β induces hepatic gluconeogenesis by enhancing FoxO1 protein stability rather than Akt-mediated FoxO1 phosphorylation and Fsk-induced CREB phosphorylation. (**A**–**C**) Mouse primary hepatocytes were infected with Ad-GFP or Ad-GADD45β for 48 h. (**A**) Cells were treated with 10 uM Fsk for 0.5 or 3 h. Western blot showing the effects of GADD45β on FoxO1 protein level and CREB phosphorylation level (**left**). Bar graph showing the ratio of pCREB to CREB and FoxO1 toHSP90 (**right**). (**B**) Cell were treated with 100 nM insulin for 0.5 or 3 h. Western blot showing the effects of GADD45β on AKT phosphorylation level and FoxO1 phosphorylation level (**left**). Bar graph showing the ratio of pAKT (Ser473) to AKT and pFoxO1 to FoxO1 quantified by ImageJ (**right**). (**C**) Western blot (**upper**) and qPCR analysis (**bottom**) showing the effects of GADD45β on FoxO1 protein and mRNA level. (**D**) Cycloheximide (CHX) chase assay showing the degradation rates of the existing FoxO1 protein. Mouse primary hepatocytes were treated with 25 ug/mL Cycloheximide (CHX) for 0.5, 1, 2, 3, or 4 h. Western blot (**upper**) and its quantification graph (**bottom**) showing FoxO1 protein levels. (**E**) Western blot and CHX chase assay showing the effects of GADD45β on FoxO1 protein stability. Mouse primary hepatocytes infected with Ad-GFP or Ad-GADD45β were treated with or without 25 ug/mL CHX for 2 h. (**F**) Luciferase assay showing effects of GADD45β on FoxO1-induced 6X-IRE promoter. HepG2 cells were co-transfected with pGL4-6X-IRE-TK and HA-FoxO1 ADA with or without Flag- GADD45β. Luciferase activity was measured 48 h after transfection and normalized to RSV β-gal levels. (**G**,**H**) qPCR analysis showing the effects of GADD45β overexpression (**G**) and KD (**H**) on IGFBP1 and NR4A1 mRNA levels in mouse primary hepatocytes. Cells infected with the Ad-GFP or Ad-GADD45β (**G**) or with the Ad-US or Ad-shGADD45β (**H**) were treated with or without 10 uM Fsk for 2 h. (**I**–**K**) HEK293 cells were co-transfected with pcDNA3-GFP-GADD45β or/and pcDNA3-RFP-FoxO1. Representative fluorescence microscopy imaging (40X objective) (**I**) and Western blot (**J**) showing intracellular localization of GADD45β and FoxO1. (**K**) Co-immunoprecipitation showing the protein–protein interaction between GADD45β and FoxO1. Data in (**A**,**F**,**G**,**H**) represent the mean ± SD (* *p* < 0.05; ** *p* < 0.005; *** *p* < 0.0005; *t*-test).

To further investigate the effects of GADD45β on Fsk-mediated CREB phosphorylation and FoxO1 protein level, mouse primary hepatocytes infected with Ad-GADD45β were treated with Fsk (Figure 3A). GADD45β overexpression increased the basal and Fsk-induced levels of FoxO1 protein, rather than Fsk-induced CREB phosphorylation (Figure 3A). On the other hand, FoxO1 is well known to be phosphorylated and inhibited by insulin-mediated phosphorylation of AKT (Figure S3A,B). However, the overexpression of KD, or KO of GADD45β did not affect insulin-mediated phosphorylation of AKT at Thr308 and Ser473 and FoxO1 at Ser256 (Figure 3B, Figure S3C–F). Therefore, we hypothesized that GADD45β would modulate gluconeogenesis by regulating FoxO1 protein stability. In mouse primary hepatocytes and HepG2 liver cells, GADD45β overexpression enhanced FoxO1 protein level without changing its mRNA level (Figure 3C, Figure S4A). Therefore, we performed the cycloheximide (CHX) protein degradation assay to investigate whether GADD45β affected FoxO1 protein stability (Figure 3D). FoxO1 protein level was gradually decreased by the treatment of CHX, a protein synthesis inhibitor, in a time-dependent manner (Figure 3D). The CHX-induced degradation of the FoxO1 protein was restored by hepatic GADD45β overexpression, suggesting the effects of GADD45β on FoxO1 protein stability (Figure 3E).

### 3.4. GADD45β Promotes Transcriptional Activity through an Increase in FoxO1 Protein Stability

Furthermore, we found that GADD45β enhanced the FoxO1-induced activities of insulin response elements (IRE) and G6PC promoter (Figure S4B,C). Therefore, we tested whether the GADD45β-enhanced protein level of FoxO1 affected FoxO1 transcriptional activity. Indeed, FoxO1 protein expression was increased when IRE promoter activities were increased by GADD45β (Figure 3F). To further emphasize the effects of GADD45β on FoxO1 transcriptional activity, we observed that hepatic GADD45β overexpression enhanced the FoxO1 target gene IGFBP1, but not the CREB/CRTC2 target gene NR4A1 (Figure 3G). Conversely, hepatic GADD45β KD suppressed the FoxO1 target gene IGFBP1, but not NR4A1 (Figure 3H). GADD45β was colocalized with FoxO1 and increased FoxO1′s protein level regardless of its cellular localization (Figure 3I,J). It physically bound to FoxO1 (Figure 3K).

### 3.5. Hepatic GADD45β is Involved in the Insulin-Mediated Reduction of Hepatic Gluconeogenic Genes

**Figure 4:**
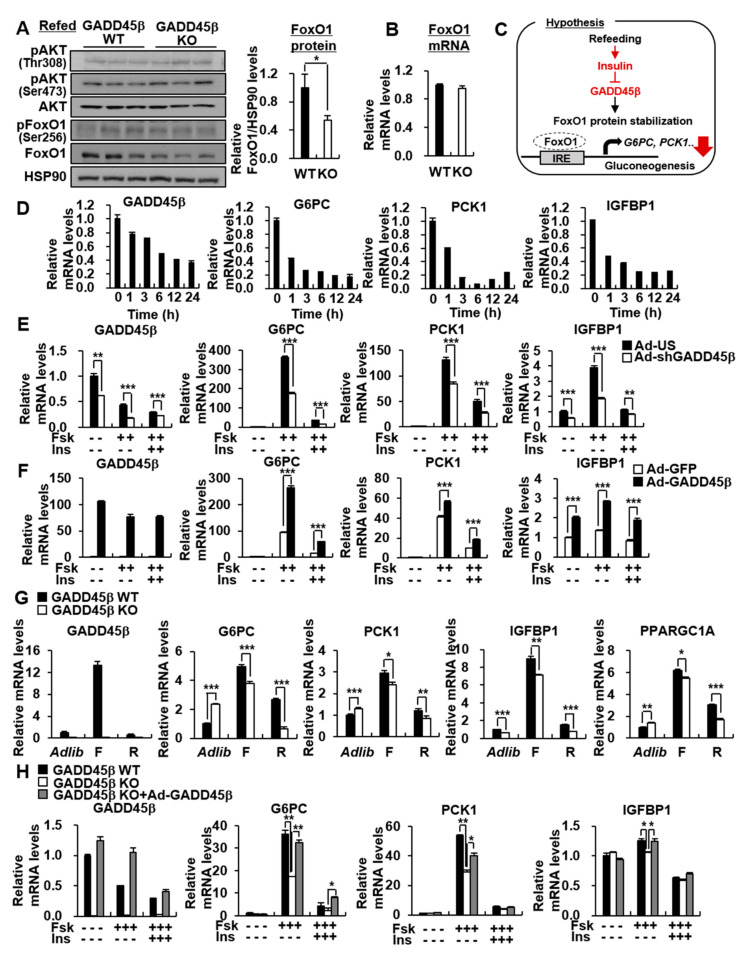
GADD45β deficiency potentiates insulin-mediated suppression of hepatic gluconeogenesis under refeeding conditions. (**A**,**B**) GADD45β WT and GADD45β KO were refed for 24 h after a 24 h fast. (**A**) Western blot analysis showing the effects of GADD45β KO on phospho- and total-protein levels of AKT and FoxO1 in the liver (**left**). Bar graph showing the ratio of FoxO1 to HSP90 quantified by ImageJ (**right**). (**B**) qPCR analysis showing FoxO1 mRNA level in the liver. (**C**) A schematic diagram showing hypothesis for the role of hepatic GADD45β in regulation of hepatic gluconeogenesis under refeeding conditions. (**D**) qPCR analysis showing the effects of insulin on GADD45β, G6PC, PCK1, and IGFBP1 mRNA levels. Mouse primary hepatocytes were treated with 100 nM insulin (Ins) at times indicated. (**E**) qPCR analysis showing the effects of GADD45β knockdown on GADD45β, G6PC, PCK1, and IGFBP1 mRNA levels. Mouse primary hepatocytes infected with Ad-US or Ad-shGADD45β were treated with or without 10 uM Fsk for 2 h and 100 nM Ins for 24 h. (**F**) qPCR analysis showing the effects of GADD45β overexpression on GADD45β, G6PC, PCK1, and IGFBP1 mRNA levels. Mouse primary hepatocytes infected with Ad-GFP or Ad-GADD45β were treated with or without 1 uM Fsk for 2 h and 10 nM Ins for 24 h. (**G**) qPCR analysis of GADD45β, G6PC, PCK1, IGFBP1, and PPARGC1A mRNA levels in livers from GADD45β WT or GADD45β KO mice under ad libitum feeding (Adlib), 24 h fasting (F), and 24 h fasted/24 h refed (R) conditions (*n* = 5/group). (**H**) qPCR analysis showing the effects of hepatic GADD45β restoration on GADD45β, G6PC, PCK1, and IGFBP1 mRNA levels. Primary hepatocytes isolated from GADD45β WT and KO mice were infected with Ad-GFP or Ad-GADD45β, and were treated with 1 uM Fsk for 1 h in the absence or presence of 10 nM Ins for 24 h. Data in (**A**,**E**–**H**) represent the mean ± SD (* *p* < 0.05, ** *p* < 0.005, *** *p* < 0.0005, *t*-test).

Hepatic GADD45β expression was decreased under refeeding conditions (Figure 1B). Further, we found that FoxO1 protein level was significantly decreased in the livers of GADD45β KO mice under refeeding conditions, without changes in phosphorylation and mRNA levels of hepatic FoxO1 (Figure 4A,B). Therefore, we hypothesized that insulin could reduce the expression of hepatic GADD45β and that the reduced expression of hepatic GADD45β might affect the insulin-mediated suppression of gluconeogenic gene expression through regulating the protein stability and transcriptional activity of FoxO1 (Figure 4C). Indeed, insulin decreased GADD45β expression concomitant with reduced gluconeogenic gene expression (Figure 4D). GADD45β KO and KD enhanced the insulin-induced suppression of the FoxO1 target IGFBP1 and gluconeogenic genes (Figure 4E, Figure S5). Conversely, hepatic GADD45β expression impeded the insulin-mediated reduction of the FoxO1 target gene IGFBP1 and gluconeogenic genes (Figure 4F), similar to the effects of GADD45β on FoxO1 transcriptional activity in the absence or presence of insulin (Figure S4C). As expected, GADD45β KO mice exhibited a more significantly reduced expression of hepatic gluconeogenic genes under the refeeding conditions than fasting conditions (Figure 4G). The restoration of GADD45β recovered its effects on gluconeogenic gene expression in GADD45β KO primary hepatocytes (Figure 4H).

## 4. Discussion

In this study, we showed that hepatic GADD45β regulates hepatic gluconeogenesis. Specifically, GADD45β regulated hepatic gluconeogenesis by increasing the protein stability and transcriptional activity of FoxO1, a master transcription factor for modulating hepatic gluconeogenesis. Blood glucose level is mainly balanced by glucagon and insulin [4], which are closely associated with two master transcription factors. Insulin lowers blood sugar levels by suppressing hepatic gluconeogenesis through the phosphorylation of FoxO1, whereas glucagon stimulates hepatic gluconeogenesis through the phosphorylation of CREB [3]. Glucagon can also enhance FoxO1 activity by regulating its protein stability [14]. Therefore, we focused on these three mechanisms as we investigated the effects of GADD45β on hepatic gluconeogenesis. We observed that GADD45β increased gluconeogenic gene expression induced by Fsk. However, GADD45β did not affect CREB phosphorylation and CRTC2/CREB-target NR4A1 mRNA levels by Fsk. On the other hand, GADD45β affected the FoxO1-target IGFBP1 mRNA levels, but not insulin-mediated FoxO1 phosphorylation. Further, we found that GADD45β expression promoted FoxO1 protein stability under basal and Fsk-stimulated conditions, resulting in increased FoxO1 transcriptional activity.

The expression of hepatic gluconeogenic genes was decreased in both whole-body KO and hepatic KD mice of GADD45β. However, the effects of GADD45β deficiency on blood glucose levels was more consistent and evident in hepatic GADD45β KD than whole-body GADD45β KO under both NCD and HFD conditions. Therefore, we focused on investigating the direct effects of GADD45β on the liver using hepatic GADD45β KD mice. On the other hand, there seems to be a difference between the two mouse models. It might be due to the difference between whole body and liver depletion. Whole-body GADD45β KO mice exhibited decreased FA transporter CD36 gene expression and increased hepatic TG levels, similar to the previous report [20]. Additionally, we observed decreased FA oxidation genes in the livers of whole-body GADD45β KO mice. In this study, GADD45β KD was similar to GADD45β KO in inhibiting hepatic gluconeogenesis, but it increased the expression of genes involved in FA uptake and FA oxidation. It might be related to the induction of FA oxidation in the AMPK-CPT1 axis [26,27], because GADD45β KD promoted the CPT1 gene expression and AMPK phosphorylation. Therefore, we confirmed GADD45β KD-induced FA oxidation by OCR measurements. Additionally, similarly to whole-body KO, GADD45β KD increased hepatic TG accumulation. The pool of FAs is very important, because it could act as precursors for gluconeogenesis [28]. However, in this study, most FAs that might have been increased in the livers of GADD45β KD mice might have been used for FA oxidation and FA synthesis rather than as precursors for gluconeogenesis [28].

Most stress-related factors, especially ER stress-related factors, are involved in the induction of gluconeogenesis [29,30,31]. Similarly, GADD45β, as a stress sensor and a fasting marker, induced hepatic gluconeogenesis. The role of GADD45β in hepatic gluconeogenesis might be related with STAT3 because STAT3 can function as a transcriptional repressor of GADD45β and the STAT3-mediated suppression of hepatic gluconeogenesis was associated with FoxO1 activity [20,21,22,23].

On the other hand, the expression of the FoxO1 target IGFPB1 and common gluconeogenic targets G6PC and PCK1 was increased at the beginning of the fasting periods. Subsequently, hepatic GADD45β expression was enhanced. Therefore, it was thought that fasting-induced hepatic GADD45β might be necessary to maintain the activity rather than to initially induce FoxO1 activity in the liver. In addition, we found that hepatic GADD45β expression was decreased by insulin, and insulin-reduced hepatic GADD45β expression was involved in insulin-mediated suppression of hepatic gluconeogenic genes. Therefore, GADD45β might be required for the metabolic adaptation to maintain blood glucose level during the fasting–refeeding cycle.

## 5. Conclusions

Our findings demonstrate that GADD45β could be an essential regulator of hepatic gluconeogenesis by modulating FoxO1 protein stability. It will deepen our understanding of the regulatory mechanism underlying FoxO1-mediated gluconeogenesis.

## Data Availability

Further information and requests for resources and reagents should be directed to and will be fulfilled by the Lead Contact, Kyoung-Jin Oh (kjoh80@kribb.re.kr).

## Supplementary Materials

The following are available online at https://www.mdpi.com/2227-9059/9/1/50/s1, Figure S1: Levels of blood glucose and expression of the hepatic glucose and lipid metabolism-related genes in livers of GADD45β knockout (KO) mice under fasting and HFD conditions. Figure S2: The effect of hepatic GADD45β knockdown (KD) on hepatic TG levels and the basal and AICAR-induced AMPK phosphorylation. Figure S3: The effects of GADD45β on the insulin-mediated phosphorylation of AKT. Figure S4. The effects of GADD45β on the protein stability and transcriptional activity of FoxO1. Figure S5: The effects of GADD45β KO on insulin-mediated suppression of hepatic gluconeogenic genes.
