# Supplementary material for: GADD45β Regulates Hepatic Gluconeogenesis via Modulating the Protein Stability of FoxO1
Source: Biomedicines. 2021 Jan 8;9(1):50. doi: 10.3390/biomedicines9010050 (PMC7827134; doi:10.3390/biomedicines9010050)
Supplement: Supplementary file 1 [file biomedicines-09-00050-s001.pdf]

Supplementary Materials for

GADD45β regulates hepatic gluconeogenesis via modulating the protein stability of FoxO1

Hyunmi Kim[1,2], Da Som Lee[1,2], Tae Hyeon An[1,2], Tae-Jun Park[1], Eun-Woo Lee[1], Baek Soo Han[1,2], Won Kon Kim[1,2], Chul-Ho Lee[3], Sang Chul Lee[1,2], Kyoung-Jin Oh[1,2,*] and Kwang-Hee Bae[1,2,*]

[1] Metabolic Regulation Research Center, Korea Research Institute of Bioscience and Biotechnology (KRIBB), Daejeon 34141, Republic of Korea; khm7607@kribb.re.kr (H.K.); dasom89@kribb.re.kr (D.S.L.); anth0291@kribb.re.kr (T.H.A.); tjpark@kribb.re.kr (T.-J.P.); ewlee@kribb.re.kr (E.-W.L.); bshan@kribb.re.kr (B.S.H.); wkkim@kribb.re.kr (W.K.K.); lesach@kribb.re.kr (S.C.L.); kjoh80@kribb.re.kr (K.-J.O.); khbae@kribb.re.kr (K.-H.B.)

[2] Department of Functional Genomics, KRIBB School of Bioscience, Korea University of Science and Technology (UST), Daejeon 34141, Republic of Korea.

[3] Laboratory Animal Resource Center, Korea Research Institute of Bioscience and Biotechnology (KRIBB), Daejeon 34141, Republic of Korea; chullee@kribb.re.kr (C.-H.L.)

* Correspondence: kjoh80@kribb.re.kr (K.-J.O.); khbae@kribb.re.kr (K.-H.B.)

Supplementary Materials

Figure S1. Levels of blood glucose and expression of the hepatic glucose and lipid metabolism-related genes in livers of GADD45β knockout (KO) mice under fasting and HFD conditions.

Figure S2. The effect of hepatic GADD45β knockdown (KD) on hepatic TG levels and the basal and AICAR-induced AMPK phosphorylation.

Figure S3. The effects of GADD45β on the insulin-mediated phosphorylation of AKT.

Figure S4. The effects of GADD45β on the protein stability and transcriptional activity of FoxO1.

Figure S5. The effects of GADD45β KO on insulin-mediated suppression of hepatic gluconeogenic genes.

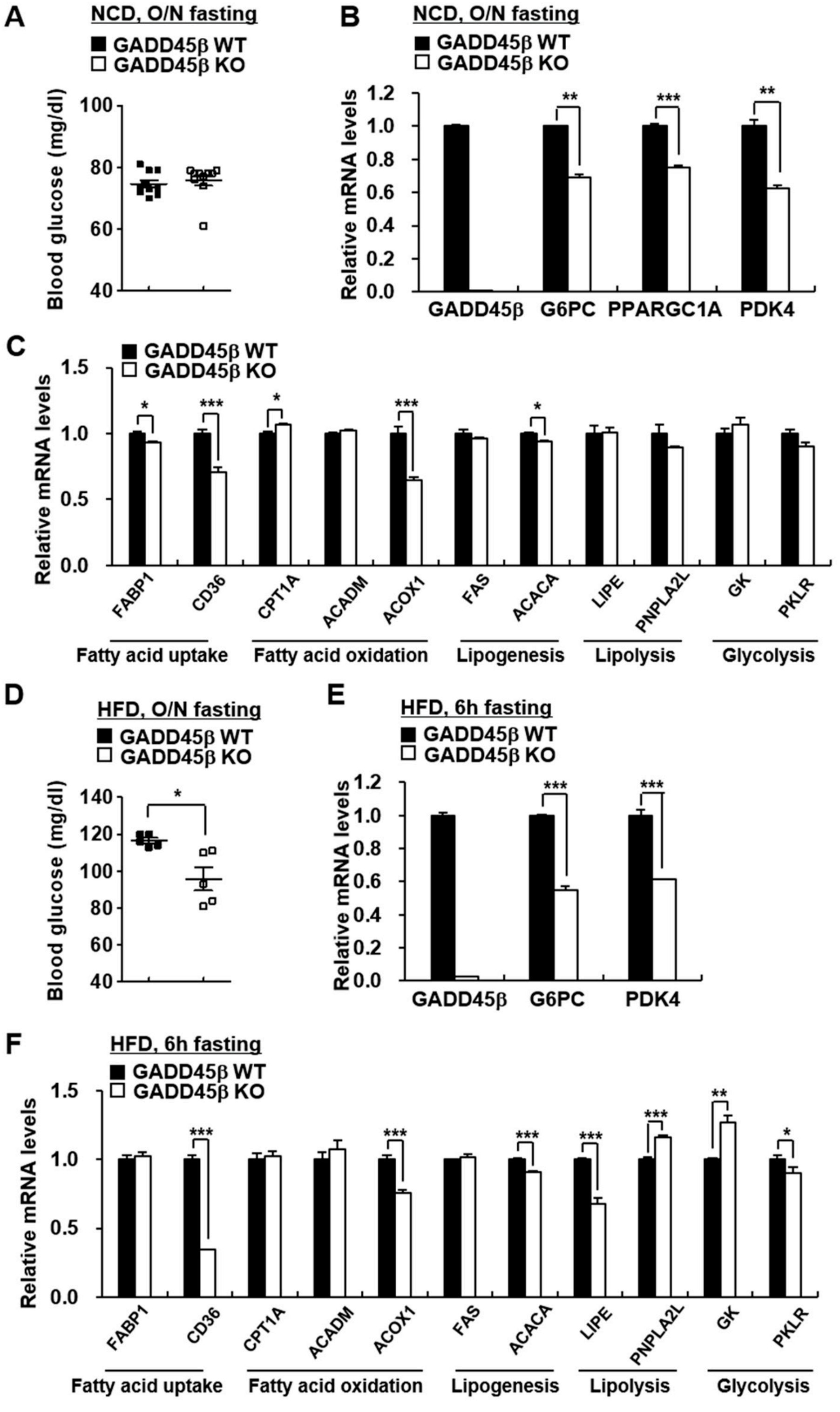

Figure S1. Levels of blood glucose and expression of the hepatic glucose and lipid metabolism-related genes in livers of GADD45β knockout (KO) mice under fasting and HFD conditions.

(**A**-**C**) 8-week-old male GADD45β WT or GADD45β KO mice were fasted for 16 h (n = 10/group). (**A**) Fasting (16 h) blood glucose levels. (**B**,**C**) qPCR analysis showing the effects of GADD45β KO on expression of the hepatic glucose and lipid-related genes in livers. (**D**-**F**) 8-week-old male GADD45β WT or GADD45β KO mice were fed a HFD for 12 weeks (n = 5/group). (**D**) Fasting (16 h) blood glucose levels. (**E**,**F**) qPCR analysis showing the effects of GADD45β KO on expression of the hepatic glucose and lipid-related genes in livers. Data in (B,C,E,F) represent the mean ± SD, and data in (A,D) represent the mean ± SEM (*$p < 0.05$; **$p < 0.005$; ***$p < 0.0005$; t-test).

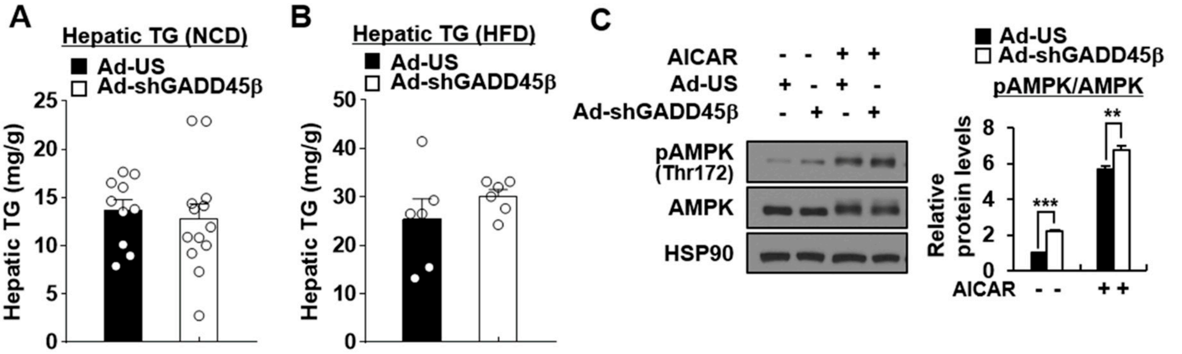

Figure S2. The effect of hepatic GADD45β knockdown (KD) on hepatic TG levels and the basal and AICAR-induced AMPK phosphorylation.

(**A**) 8-week-old C57BL/6 male mice were infected with Ad-shGADD45β ($n = 15$) or Ad-US control ($n = 10$) for 7 days. Hepatic triglyceride (TG) levels under 16-h fast conditions. (**B**) 8-week-old C57BL/6 male mice fed a HFD for 12 weeks were infected with Ad-shGADD45β ($n = 6$) or Ad-US control ($n = 6$) for 7days. Hepatic triglyceride (TG) levels under 16 h fast conditions. (**C**) Mouse primary hepatocytes infected with Ad-US or Ad-shGADD45β were treated with 1 mM AICAR for 2 h. Western blot showing the effects of GADD45β KD on the phosphorylation level of AMPK. Data in (**C**) represent the mean ± SD (*$p < 0.05$; t-test).

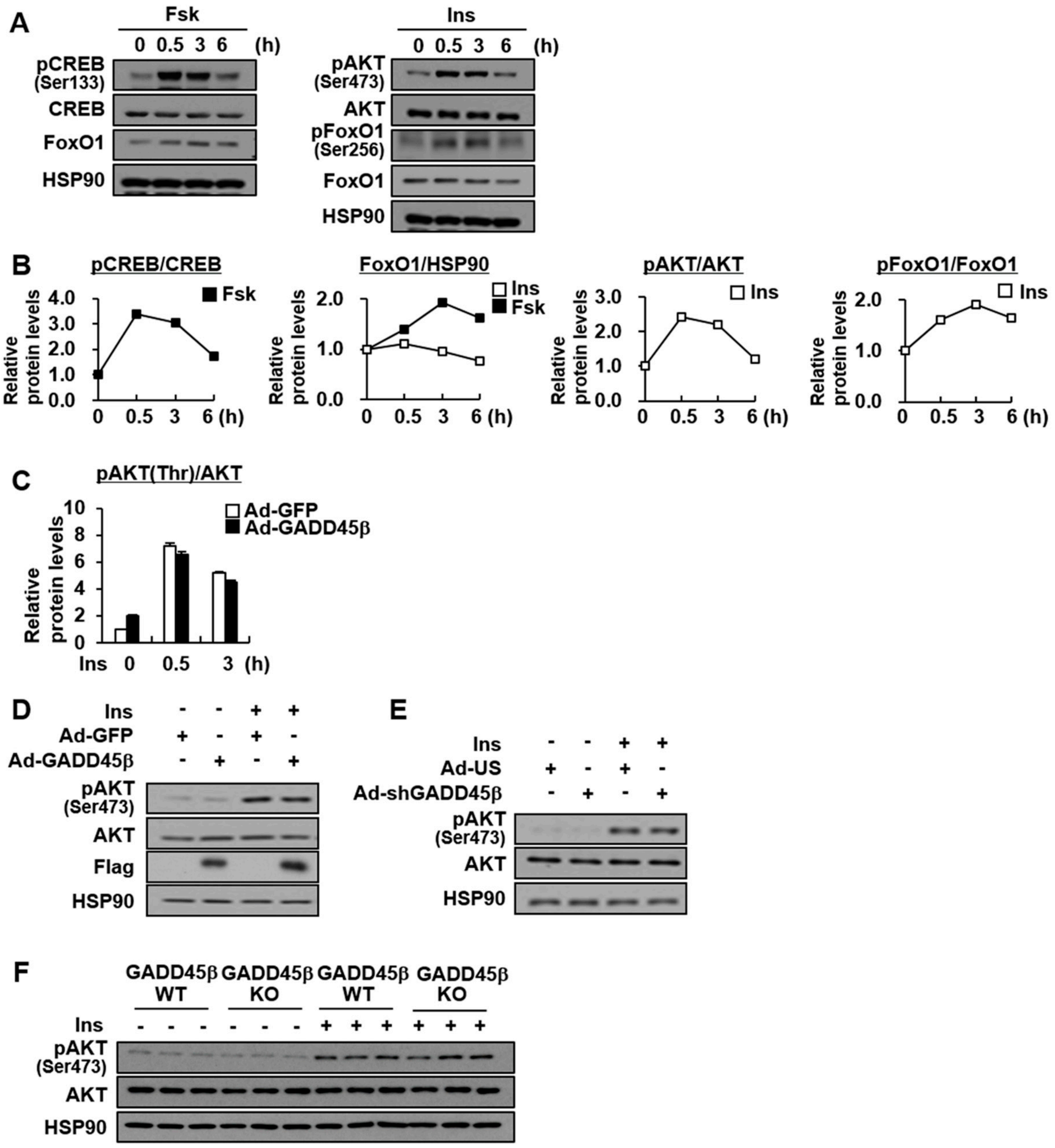

Figure S3. The effects of GADD45β on the insulin-mediated phosphorylation of AKT.

(**A**,**B**) Western blot (**A**) and quantified graph (**B**) showing the effects of Forskolin (Fsk) and insulin (Ins) on phosphor- and total protein levels of CREB, FoxO1, and AKT. Mouse primary hepatocytes were treated with 10uM Fsk or 100 nM Ins for 0, 0.5, 3, or 6 h. (**C**) Cell were treated with 100 nM insulin for 0.5 or 3 h. Bar graph showing the ratio of pAKT (Thr308) to AKT. (**D**-**F**) Western blot showing the effects of GADD45β overexpression (**D**), KD (**E**), and KO (**F**) on phosphor- and total levels of AKT. Mouse primary hepatocytes were infected with Ad-GFP or Ad-GADD45β (**D**) or with Ad-US or Ad-shGADD45β (**E**). Cells were treated with 100 nM Ins for 10 min.

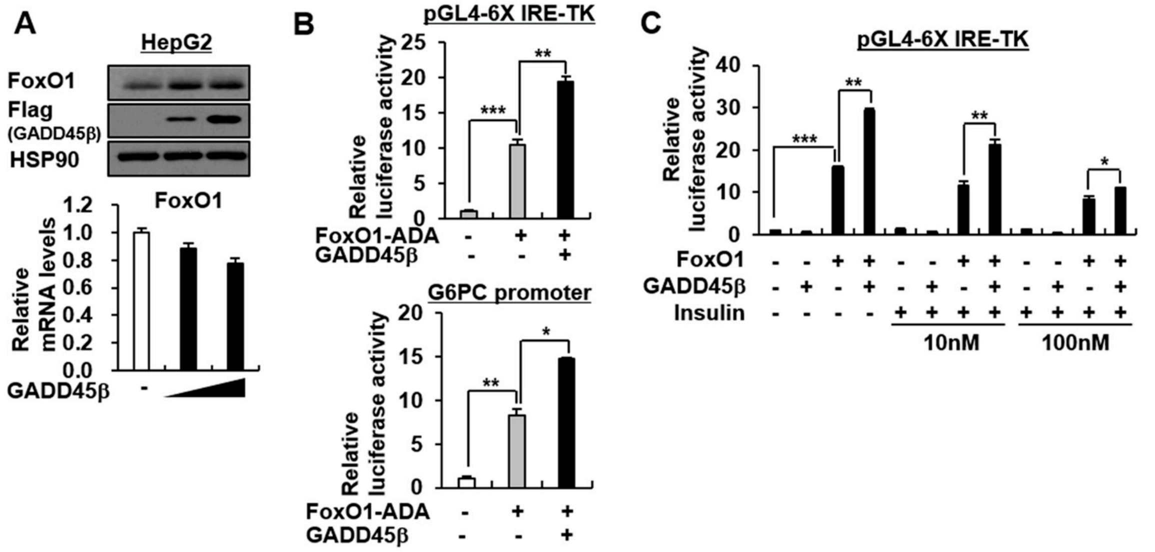

Figure S4. The effects of GADD45β on the protein stability and transcriptional activity of FoxO1.

(**A**) Western blot (upper) and qPCR analysis (bottom) showing the effects of GADD45β on FoxO1 protein and mRNA level. HepG2 cells were transfected with Flag- GADD45β. (**B**) Luciferase assay showing effects of GADD45β on FoxO1-induced promoter activities of 6X insulin response element (IRE) and G6PC. HepG2 cells were co-transfected with pGL4-6X-IRE-TK (upper) or pGL4-G6PC (bottom) promoters and HA-FoxO1 ADA with or without Flag- GADD45β. (**C**) Luciferase assay showing effects of GADD45β on insulin-suppressive effects of 6X insulin response element (IRE) promoter. HepG2 cells were co-transfected with pGL4-6X-IRE-TK (upper) promoters and HA-FoxO1 ADA with or without Flag- GADD45β, and treated with 10 nM or 100nM insulin for 10 min. Luciferase activity was measured 48 h after transfection and normalized to RSV β-gal levels. Data in (**B**,**C**) represent the mean ± SD (*$p < 0.05$; **$p < 0.005$; ***$p < 0.0005$; t-test).

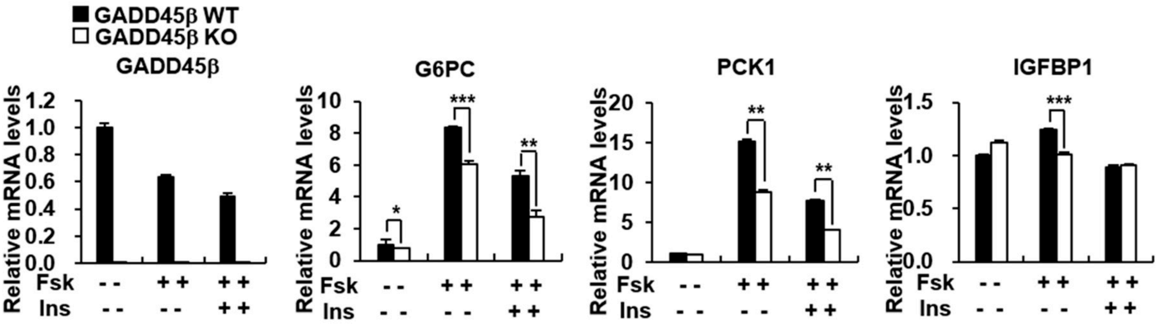

Figure S5. The effects of GADD45β KO on insulin-mediated suppression of hepatic gluconeogenic genes.

qPCR analysis showing the effects of GADD45β KO on GADD45β, G6PC, PCK1, and IGFBP1 mRNA levels. Mouse primary hepatocytes were treated with or without 10 uM Fsk for 2 h in the absence or presence of 100 nM Ins for 24 h. Data represent the mean ± SD (*$p < 0.05$; **$p < 0.005$; ***$p < 0.0005$; t-test).
